# Swedish snus and the GothiaTek^® ^standard

**DOI:** 10.1186/1477-7517-8-11

**Published:** 2011-05-16

**Authors:** Lars E Rutqvist, Margareta Curvall, Thord Hassler, Tommy Ringberger, Inger Wahlberg

**Affiliations:** 1 Scientific Affairs Group, Swedish Match AB, Maria Skolgata 83, 118 85 Stockholm, Sweden; 2 Chemical Analysis, Research & Development Group, Swedish Match North Europe AB, Maria Skolgata 83, 118 85 Stockholm, Sweden; 3 Research & Development Group, Swedish Match North Europe AB, Maria Skolgata 83, 118 85 Stockholm, Sweden

**Keywords:** smokeless tobacco, Swedish snus, history, chemical analysis, epidemiology

## Abstract

Some smokeless tobacco products, such as Swedish snus, are today considered to be associated with substantially fewer health hazards than cigarettes. This risk differential has contributed to the scientific debate about the possibilities of harm reduction within the tobacco area. Although current manufacturing methods for snus build on those that were introduced more than a century ago, the low levels of unwanted substances in modern Swedish snus are largely due to improvements in production techniques and selection of raw materials in combination with several programs for quality assurance and quality control. These measures have been successively introduced during the past 30-40 years. In the late 1990s they formed the basis for a voluntary quality standard for Swedish snus named GothiaTek^®^. In recent years the standard has been accepted by the members of the trade organization European Smokeless Tobacco Council (ESTOC) so it has now evolved into an industrial standard for all smokeless tobacco products in Europe.

The initial impetus for the mentioned changes of the production was quality problems related to microbial activity and formation of ammonia and nitrite in the finished products. Other contributing factors were that snus came under the jurisdiction of the Swedish Food Act in 1971, and concerns that emerged in the 1960s and 1970s about health effects of tobacco, and the significance of agrochemical residues and other potential toxicants in food stuffs.

This paper summarizes the historical development of the manufacture of Swedish snus, describes the chemical composition of modern snus, and gives the background and rationale for the GothiaTek^® ^standard, including the selection of constituents for which the standard sets limits. The paper also discusses the potential future of this voluntary standard in relation to current discussions about tobacco harm reduction and regulatory science in tobacco control.

## Introduction

The term smokeless tobacco (ST) includes a broad range of products that vary considerably with regard to composition and content of potential toxicants [1,2]. Consequently, there is also a large variability with regard to health effects (this concept is sometimes referred to as "continuum of risk"). The Royal College of Physicians in London stated that *"smokeless tobacco is 10-1,000 less hazardous to health than smoking, depending on the product" *[3]. In line with this statement, the WHO Tobacco Regulatory Committee (TobReg) recently concluded that *"Among the smokeless tobacco products on the market, products with low levels of nitrosamines, such as Swedish snus, are considerably less hazardous than cigarettes..." *[2].

The health effect profile of Swedish snus and the ability of snus to replace cigarettes among smokers has been evidenced by numerous epidemiological studies [4-6]. Cultural factors have probably contributed to the observed usage patterns. However, the most important determinants of health effects among individual users are the chemical properties of the product. The manufacture of Swedish snus has in principle remained the same since the early 1800s. However, as snus became more popular in Sweden during the late 1960s and came under the jurisdiction of the Swedish Food Act in 1971, the state-owned company responsible for snus production at the time modernized the production techniques and introduced several programs for quality assurance and quality control. This later formed the basis for a comprehensive quality standard for Swedish snus named GothiaTek^®^.

The current paper is a summary of the background and rationale for these developments together with a description of the production and properties of modern, Swedish snus, a product category that is central to current discussions about the possibilities of harm reduction in the tobacco area. Although the GothiaTek^® ^standard sometimes is referenced in the scientific literature, no authoritative publication describing it has previously been published.

### Definition of Swedish snus

According to the trade organization European Smokeless Tobacco Council (ESTOC), "snus" is defined as a ST product for oral use "traditionally produced and used in Sweden ... the manufacturing process is a heat treatment process" [7].. This definition distinguishes snus from all other types of ST including some products recently introduced on the North American market which have distinctly different characteristics [8].

The 2009 WHO TobReg report underscored that *"the differences in risks associated with use of different smokeless tobacco products mean that it would be scientifically inappropriate to consider smokeless tobacco as a single product for the purposes of estimating risk or setting policies"*. Hence, it is critical to use clear definitions of ST products when discussing risk differentiation. In this paper we refer to Swedish snus by the ESTOC definition and not any wider, arbitrary definition.

### History of snus

Swedish snus was invented during the early 1800s. It was made of ground tobacco leaves, water, salt, and potash [9]. Ease of use during manual work compared to smoked tobacco and a low cost probably contributed to its popular appeal. Traditionally the snus pinch was placed between the gum and the anterior part of the upper lip, that is, at a distance from the orifices of the large salivary glands. This meant that there was no excessive saliva mixed with the tobacco, which obviated the need for spitting, a habit typically associated with some other forms of ST usage.

**Figure 1:**
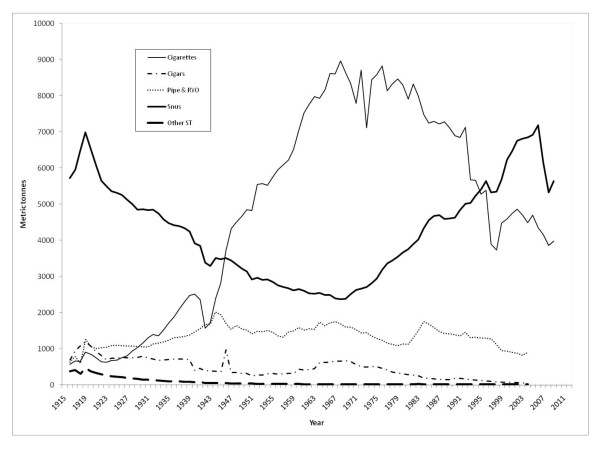
**Tobacco sales in Sweden 1916-2008 according to product category**. The data refer to estimates of delivered quantities to points of sale with adjustment for recalls and returns. Available official records do not permit adjustments for tax-free or cross-border trading, nor for illegal entries into or out of the country. RYO: "Roll-your-own" tobacco, ST: smokeless tobacco [37-39].

Until the early 1940s snus was the predominant form of tobacco used in Sweden (Figure 1). During the 1930s cigarettes gained popularity, and eventually replaced snus as the most commonly used tobacco product, an epidemic similar to that in most western countries during the 20^th ^century.

In the 1960s the Royal College of Physicians [10] and the U.S. Surgeon General [11] published reports that linked smoking to lung cancer. This contributed to increased societal smoking control efforts and a shift in tobacco habits that started during the late 1960s: a decline of cigarette smoking and an increase in use of snus (Figure 1).

Although snus has never been marketed as a smoking cessation aid, many Scandinavian smokers have replaced cigarettes with snus [6,12]. In population-based surveys, snus is the most frequently reported cessation aid, and use of snus is associated with higher rates of sustained quitting than other methods, such as, nicotine replacement therapy [5,6,13]. However, the role of snus for smoking cessation remains controversial. Critics have pointed out the observational character of the available data, and the lack of controlled clinical trials.

Snus has always been the predominant form of smokeless tobacco in Sweden. Other types, such as, chewing tobacco have always constituted less than one per cent of the total sales of ST (Figure 1).

Since the formation in 1915 of the Swedish state-owned tobacco monopoly (dismantled during the 1960s) snus has been produced by essentially one company, either the Tobacco Monopoly, or its successors: the state-owned company (STA) which was transformed during the 1990s into the current private company Swedish Match. The first competitor appeared in 1992, and today there are several manufacturers of snus in Sweden. However, Swedish Match retains a market share of c.85%.

### Production methods

#### The early years

Documents in the archives of the Swedish Tobacco Museum include recipes for snus production dating back to the early 1800s. One of the oldest is the formula for "The Making of Ljunglöf's well-reputed Snus" [14] which prescribes a mixture of Swedish and American tobacco. The tobacco was ground into a powder which was sifted in a rotating metal drum. One batch consisted of 144 kg of leaf powder. A total of 133 liters of water were used for moistening, plus 2.3 liters of table salt. The powder, salt and water were mixed and put into wooden cases to stand for six days in a warm oven. After that 15 kg of potash was mixed in, and the snus was cooled and became ready for use.

Documents in the Tobacco Museum archives reveal that recipes and production techniques used by other manufacturers at the time were similar to Ljunglöf's. In particular, they also used variations of the heat treatment technique [9]. The temperatures used typically ranged from 50 to 80 degrees Centigrade. Most manufacturers used flavorings, such as, salmiac, pepper, and lemon or bergamot oil [I. Junhem, personal communication].

During the 20^th ^century production of snus became more centralized. In the late 1960s snus was only produced in one factory using almost the same recipes and manual production techniques as during the 1800s, although the temperature during the heat treatment had been raised slightly to decrease problems related to microbial contamination.

#### Research & development since the 1970s

With the growing popularity of snus in the late 1960s, several quality problems were noted with the old, manual production techniques. During storage ammonia was sometimes formed in the finished products resulting in an uncontrolled increase in pH. The exact chemical mechanisms behind this phenomenon were largely unknown. This prompted a research and development program aimed at finding methods to deal with the quality problems with the old techniques.

The most important outcome was the introduction of a modernized manufacturing process at a newly built factory in 1982. Another impetus for the quality development was that snus came under the jurisdiction of the Swedish Food Act in 1971. The rationale was that it is consumed in the mouth, and, therefore, is partly ingested. Compliance with the Food Act implied stricter hygienic requirements and restrictions as to the range of allowed ingredients, additives, and containers, all of which must be food-grade.

As a result of collaboration with the Swedish Food Authority several initiatives for quality assurance and quality control were successively introduced in the production during the 1970s through the 1990s. This collaboration was in part a response to the scientific and public debates about health effects of tobacco products in general, and, more specifically, the role of potential toxicants and agrochemical residues in smokeless tobacco products - which paralleled a similar debate concerning food stuffs. The routine monitoring of the chemical properties of snus was greatly expanded; assays of tobacco-specific nitrosamines (TSNAs) were introduced in 1984, and an extensive, annual chemical testing of all snus brands started in 1988.

These initiatives and programs subsequently formed the basis for a voluntary, comprehensive quality standard named GothiaTek^®^. It was first announced on a company web-site in 2000, although the components of the standard had been phased into the routine production since the early 1980s [15].

#### Problems related to microbial activity

Research during the 1970s revealed that the main cause of the quality problems with snus was related to microbial growth, either because of residual bacterial activity after the heat treatment or bacterial contamination during the old, manual production process. This open manufacture was also sensitive to contamination with moulds, particularly if pH was below c. 8.

Toxicologists at the Swedish Food Authority noted in the mid 1970s that snus occasionally could contain high levels of nitrite, probably due to growth of nitrate-reducing bacteria. Research about health effects of nitrite and its relation to the formation of potentially carcinogenic nitrosamines had started already in the 1960s and was widely publicized in the 1970s and 1980s.

Several measures were tested to reduce the microbial activity in snus, to prevent the formation of nitrite, and to improve the stability of the finished product, while at the same time not compromising consumer acceptance. Eventually, the optimal approach was found to be a combination of a modified production technique based on processing of the ground tobacco in closed process blenders at much higher temperatures than previously, pH stabilization with sodium carbonate, and use of humectants to lower the water activity in the finished product. Products with a high water activity tend to support more microorganisms.

The new production methods resulted in a finished product with virtually no microbial activity, and a much improved, and consistent stability. Consumer testing among individuals used to the "old" snus products also indicated a high level of acceptance.

#### Modern production of snus

A blend of leaf tobaccos is ground and sieved to specified particle sizes. The ground tobacco is mixed with water and sodium chloride in closed process blenders. The mixture is then subjected to a computer-controlled heat-treatment process, the purpose of which is to improve the taste and to reduce microbial activity to obtain a proper shelf life. The heating is achieved by using hot water and by injecting steam into the blenders. It involves temperatures up to 80-100 degrees centigrade during several hours. Finally the snus is cooled and other ingredients such as flavors and humectants are added before the finished product is fed to automatic packers.

Retailers are encouraged to keep the snus refrigerated in order to meet the criteria for the "best-before" labeling. However, there is no need for a completely uninterrupted cool chain as the low bacterial activity in the product implies stability also at room temperature.

#### "Snus ageing"

**Figure 2:**
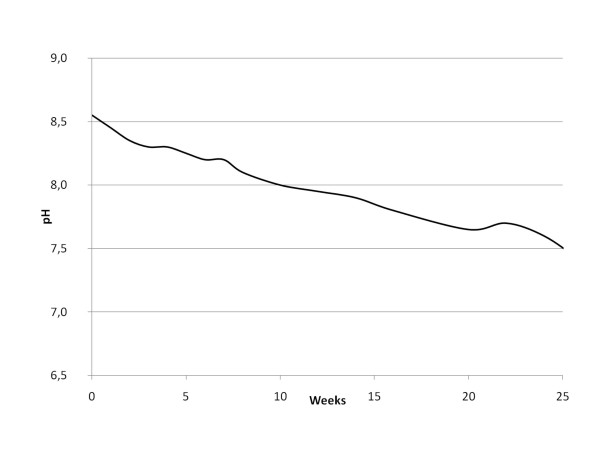
**Natural decline of pH in snus (pouch products) manufactured according to the GothiaTek^® ^standard during storage at 20 degrees centigrade (data based on internal Swedish Match documentation)**.

It has always been known that snus has a limited shelf life and in this regard suffers from similar problems as most other food stuffs with a high water content. However, before the introduction of the modified production techniques in 1982, the most prominent problems with snus quality were related to microbial activity, formation of nitrite (and, hence, probably TSNA formation), and an uncontrolled increase in pH. Snus produced with the modern techniques has a very low microbial activity and is thus much more stable. Today, ageing implies reduced water content, and a consequent loss of perceived product freshness, decrease in pH (related to a combination of oxidation of tobacco constituents and evaporation of volatile amines, including ammonia) and a decrease in nicotine content also due to oxidation. Figure 2 shows the natural decline of pH over time at room temperature in modern snus. Nitrosamines are not formed in the container during storage, even if kept at room temperature [16]. Snus ageing can be slowed by keeping the product refrigerated below 8 degrees centigrade. Ageing is more or less halted if the product is deep frozen.

### Chemical composition of snus

The components of Swedish snus are a blend of air and/or sun-cured tobacco, water, sodium chloride, sodium carbonate, humectants, and flavoring agents.

The water content is around 45-60%. Sodium chloride adds flavor and prevents microbial activity. Sodium carbonate helps to stabilize the pH. The target pH level in the production of most traditional snus brands is about 8.5 with some batch to batch variation due to natural variation of the raw tobacco. Before the 1970s the pH of snus was typically higher, but with the introduction of new production technique, the target pH level was intentionally reduced as a result of research on "snus lesions" in the gingival and buccal mucosa which were found to be related to pH level [17]. Abrasions of the buccal mucosa were at the time hypothesized to increase the absorption of potential toxicants. Traditionally potassium carbonate was used instead of sodium carbonate. The switch was made for technical reasons: sodium carbonate is more water soluble and using a water solution facilitates the production.

Humectants, such as, propylene glycol and glycerol, serve two purposes; to retain moisture, and lower the water activity.

Snus is typically flavored using food approved flavors to achieve the desired brand characteristics. In part, flavoring is used to compensate for the fact that fire-cured tobacco is no longer used in snus (see section on "The GothiaTek^® ^standard").

Swedish snus does not contain added sugars. The low sugar content (<2%) comes solely from the tobacco.

#### Constituents

There are several thousands of different constituents in natural, raw tobacco [18]. The number is probably similar to that found in other plants and in common food-stuffs. Major classes include: primary plant metabolites such as amino acids and proteins, carbohydrates, lignins, fatty acids, and degradation products from chlorophyll. Raw tobacco also contains secondary metabolites such as nicotine and related alkaloids, flavonoids, and polyphenols, as well as isoprenoids and degraded isoprenoids. Contaminants taken up from the soil, agrochemicals, or air-borne pollution include heavy metals, and radiochemicals.

About 40-50 compounds with possible health significance may be found in smokeless tobacco products including N-nitrosamines (particularly the tobacco-specific nitrosamines, TSNAs), volatile aldehydes, polyaromatic hydrocarbons (PAHs), heavy metals (e g cadmium, arsenic, nickel, chromium, lead), and radioisotopes (e. g. Polonium-210).

Tobacco also contains substances that are potentially antimutagenic and anticarcinogenic including ubiquinione, alpa-tocopherol, flavonoids, isoprenoids, and certain fatty acids [19-22]. It has not been determined if the concentrations of these compounds in Swedish snus products are sufficient to provide protective effects.

### The GothiaTek^® ^standard

The standard was based on the mentioned research and development efforts dating back to the early 1970s. The different components were phased into the production during the 1980s and 1990s. However, it was not until the late 1990s that the entire production was fully consistent with the standard [15].

The principal components of the standard are:

• Constituent standard

○ Maximum levels (MLs) for selected, undesired constituents in the finished products (see Table 1)

○ Guidance Residue Levels (GRLs) for agrochemical residues in finished products

• Manufacturing standard

○ A standard for selection of raw materials:

■ Leaf tobacco selection and an "early warning" chemical analysis program designed so that the limits for undesired constituents in finished products are met.

■ An ingredient policy consistent with the Swedish Food Act for additives and flavorings.

○ Requirements for the manufacturing process.

■ Tobacco comminuted in a controlled process satisfying the requirements for specific particle size distributions.

■ Controlled heat treatment that reduces the natural microbial flora of the tobacco to specified residual limits.

■ Manufacturing in a closed system to prevent the product from being contaminated by e.g. external microflora

■ Hygienic conditions complying with the Swedish Food Act

• Consumer information:

○ Consumer package labeling including a best before date, recommended storage conditions, and a declaration of ingredients in accordance with requirements for labeling of processed food stuffs

○ A public web-site with detailed information on brand-specific product characteristics, and updated summaries of research results on health effects of snus.

**Table 1:** Maximum Levels (MLs) for unwanted substances in Swedish snus according to GothiaTek^® ^(dry weight), and average observed levels in Swedish Match's snus products 2009 (data based on internal Swedish Match documentation)

Substance	GothiaTek^® ^ML	Average level 2009
Nitrite	7.0 ppm	2.0 ppm
TSNAs (total)	10.0 ppm	1.6 ppm
N-Nitrosodimethylamine (NDMA)	10.0 ppb	0.7 ppb
Benzo(a)pyrene (B(a)P)	20.0 ppb	1.1 ppb
Lead	2.0 ppm	0.3 ppm
Cadmium	1 ppm	0.6 ppm
Arsenic	0.5 ppm	0.1 ppm
Nickel	4.5 ppm	1.3 ppm
Chromium	3.0 ppm	0.8 ppm

ppm: parts per million (corresponding to μg/g)

ppb: parts per billion (corresponding to ng/g)

#### Constituent standard

While TSNAs are specific to tobacco, the remaining constituents covered by the standard are also found in many food stuffs.

The selection of analytes with defined MLs in the standard reflects in part the quality problems that were detected during the 1970s before the modified production techniques for snus were introduced, for instance, nitrite formation.

In a report from the U.S. Department of Health published in 1986 it was suggested that the most important carcinogenic substances found in ST products were N-nitrosamines, polyaromatic hydrocarbons (PAHs), and radioisotopes (particularly Polonium-210) [23]. MLs were therefore defined for TSNAs and B(a)P as these were considered to be the most important compounds from a carcinogenic perspective in their respective class.

Limits for radioisotopes were not included in GothiaTek^® ^as the effective radiation dose from Polonium among habitual snus users was estimated to be comparable to that from the natural background radiation sources or dental x-rays [24].

As snus is regulated under the Swedish Food Act, it was considered reasonable to include lead, cadmium, nickel, and arsenic as these compounds were known to be present in many leafy vegetables (including tobacco), as they were known to have potential health effects, and as limits were defined in the Food Act for selected food stuffs. However, the Act has never set limits for constituents in smokeless tobacco products, with the exception of lead for which the ML is 3 μg/g.

In selecting the constituents to be included in the standard, consideration was also given to a list published by Hoffman & Djordjevic in 1997 [25]. Their list is more extensive than the U.S. Department of Health list from 1986 and includes some constituents that were not included in GothiaTek^®^, such as alpha/beta-angelica lactone, coumarin, hydrazine, volatile aldehydes, and ethyl carbamate (urethane). The main reasons for exclusion of these additional analytes were that they were found to be non-detectable or present only in trace amounts in snus, that robust analytical methods were unavailable at the time, or that technical developments of the production were not expected to result in decreased levels of the constituent.

The MLs defined by GothiaTek^® ^were pragmatic and based on considerations of what could be consistently achieved in large-scale production, comparisons with levels observed in common food stuffs, comparisons with estimated total daily exposure from food and drinking water, and from the observations in Swedish epidemiological studies of no increased risk of oral cancer associated with long-term use of Swedish snus with a TSNA and B(a)P content substantially higher than the defined limits [26,27].

Aside from GothiaTek^® ^no voluntary standard for ST products has been introduced that include MLs for potential toxicants. Nevertheless, quality development by several manufacturers has resulted in decreased levels of nitrosamines in many traditional brands of American moist snuff and other ST products on the North American market. However, as a result of a persistent use of fire-cured tobacco, levels of B(a)P have typically remained relatively high except in some novel products marketed as "snus" [28].

#### The Swedish Match agrochemical policy

In short, this policy requires that the contribution of agrochemical residues (pesticides, fungicides, and herbicides) from 25 g of snus shall not exceed 2.5% of the defined acceptable daily intake (ADI) according to European Union regulations. Acceptable levels may be set lower if implied by e.g. a legislated ML in any country for the agrochemical in question. Lower levels are also set if implied by levels set by "The Cooperation Centre for Scientific Research Relative to Tobacco" (CORESTA).

#### Raw tobacco selection

**Figure 3:**
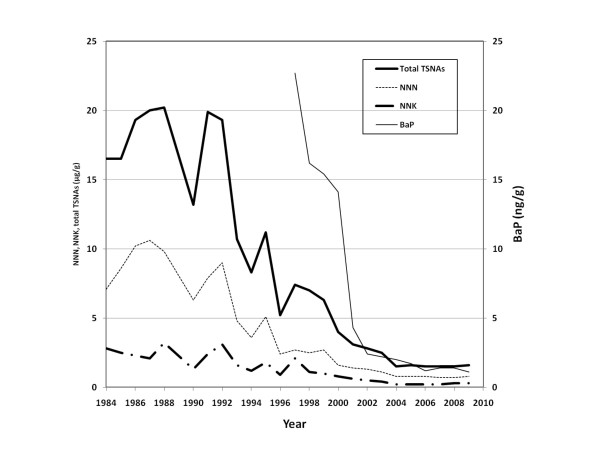
**Average levels of NNN (N'-nitrosonornicotine), NNK (4-(methylnitrosamino)-1-(3-pyridyl)-1-butanone), total TSNAs, and B(a)P (dry weight) in Swedish Match's snus products 1984-2009 (data based on internal Swedish Match documentation)**.

Traditionally snus was made from a selection of tobacco varieties typically including a large proportion of fire-cured tobacco. This resulted in relatively high levels of PAH:s in the finished products. During the 1990s use of fire-cured tobacco was phased out. Consequently, over a period of a few years, the level of B(a)P in finished snus products decreased from c. 20-25 ng/g to less than 2-3 ng/g based on dry weight (Figure 3). Fire-cured tobacco adds a distinct flavor, but the traditional flavor characteristics were preserved using food-approved flavorings.

In the early 1980s it was discovered that the main source of TSNAs in snus was certain varieties of leaf tobacco [29]. TSNAs are formed in the leaves during curing and typically increase if the tobacco is fermented. The levels may also increase in finished products as a result of nitrate-reducing microbial activity leading to formation of nitrite. The nitrate content of raw tobacco is dependent on local growing conditions and may increase, for instance, if the plants are excessively fertilized.

This awareness started research to clarify the mechanisms of TSNA formation during air-curing of tobacco, both within the company and in cooperation with university scientists with expertise in agronomy. The findings were evaluated in experiments involving seed selection, modified cultivation and curing methods [30-32]. It was also secured that the heat treatment did not lead to increased levels of TSNAs.

Curing of tobacco is essential to achieve desired flavor characteristics. Today, Swedish snus manufactured according to GothiaTek^® ^is exclusively made from selected, low-nitrosamine raw tobacco (N. tabacum) that has been air or sun-cured. This means that curing is achieved without artificial sources of heat and humidity. The curing involves complex physical and chemical changes that occur as the tobacco leaves loose moisture. Because these changes do not occur under a prescribed set of temperature and humidities, and because the chemical composition of the leaves are dependent on the climate and other growing conditions, the end product may differ considerably from one location to another and from year to year. This helps to explain why the composition of the raw tobacco used in modern snus production varies from year to year.

To achieve the constituent limits set by GothiaTek^®^, a program was implemented based on collaboration with local growers with regard to use of seeds, agrochemicals and production techniques. An "early warning" system was also introduced including multiple sampling and chemical analyses of the tobacco, and specifications for approval before shipment. As a result, the TSNA content in finished snus products has decreased continuously and substantially since routine analyses were initiated in 1984. When the testing started the observed average levels were about 15-20 μg/g based on dry weight, compared to c. 1-2 μg/g during recent years (Figure 3).

### Chemical properties versus epidemiology

Numerous analytical epidemiological studies have been published that have looked at health effects of Swedish snus [33]. In practice, such studies invariably concern exposure to the type of snus products that were on the market many years ago. For instance, in one population-based case-control study of head-neck cancer conducted in the early 1990s, the average duration of use among those who were ever snus users was c. 20 years [26]. This means that the typical user started using snus in the early 1970s. A recent publication based on the Swedish Construction Worker Cohort [34] presented detailed information on duration of snus usage among more than 40,000 snus consumers; at inclusion in the study (1971-75 and 1978-1992), 64% reported use during 1- 9 years, 28% during 10-24 years, and 8% reported use during more than 25 years. This implies that almost two thirds of the snus users in this cohort started their snus habit during the 1970s or later.

These data make it evident that some of the epidemiological documentation about the health effects of Swedish snus relate to products manufactured before the era of the GothiaTek^® ^standard. Consequently, the snus users in these studies were exposed to products with substantially higher levels of e.g. TSNAs and B(a)P than is the case today. Theoretically, it is possible that the lowered levels of toxicants in "modern" snus imply that the health effect profile of snus has improved but this hypothesis awaits epidemiological confirmation.

## Conclusions

The first impetus for the work that later formed the basis for the GothiaTek^® ^standard was problems related to microbial activity and poor product quality. However, as a result of snus being regulated under the Swedish Food Act since 1971, and the emerging scientific debate about the content of potential toxicants in tobacco products, there was also an early recognition of the need to decrease the levels of a range of undesired substances in snus. Collaboration between toxicologists at the Swedish Food Authority and the company responsible for all snus production at the time was pivotal for the development of the standard and was facilitated by the fact that both institutions belonged to the Swedish state.

GothiaTek^® ^has been accepted by the members of the trade organization European Smokeless Tobacco Council (ESTOC). It has thus become a voluntary standard for all smokeless tobacco products in Europe. The standard formed the basis for ESTOC's recent proposal to the European Union health authority (the directorate general for health and consumer protection, DG SANCO) for a science-based regulation of smokeless tobacco products within the EU.

The selection of constituents regulated in GothiaTek^® ^and the corresponding MLs were pragmatic; the standard included the toxicants that, at the time, were considered to be the quantitatively most important for the toxicity of ST products, for instance, TSNAs and B(a)P, but excluded some on account of unavailability of robust analytical methods (e.g. volatile aldehydes). The most important criterion for the selection of toxicants was the toxicity evidence, but the MLs were pragmatic and largely based on considerations of what could technically be achieved in large-scale, routine production, although it was noted that typically this meant levels and/or exposures that were comparable or even lower than from many common food stuffs. The decision not to include radioisotopes, such as Polonium-210, in the standard was based on considerations that could be described as "hazard prioritization"; absorbed radiation doses among habitual snus users were estimated to be comparable to those from the natural background radiation.

The GothiaTek^® ^standard reflects the toxicological science and production techniques of the 1990s; the toxicant levels achieved today in routine production are lower, or much lower, than the MLs defined by GothiaTek^® ^(Table 1). Techniques for chemical analysis have improved. Also, an improved scientific base now exists for formal toxicological risk assessments of different tobacco products. These circumstances suggest that it is now appropriate to revisit the MLs according to GothiaTek^® ^as well as the selection of regulated constituents. The standard should also be updated based on a modern risk assessment approach.

The WHO Framework Convention for Tobacco Control (FCTC) recognizes the need for tobacco product regulation [35]. As a result, the WHO TobReg has published several reports to provide a scientific foundation for such regulation. A recent paper summarized a proposal developed by a joint IARC and WHO working group for performance standards for cigarettes and a strategy to use them to mandate a reduction in the toxicant yields for cigarette smoke [36]. There are obviously major differences between regulating cigarettes compared to ST products. However, some basic principles suggested by the report might be applicable also to ST products: initiation of policy change beginning with annual reporting of selected toxicant levels from manufacturers to regulatory authorities, followed by the promulgation of MLs based on what may be achievable with new technology or product designs, selection of toxicants from comprehensive lists including those compounds thought to play a major role for product toxicity, prioritization within those lists based on available animal and human toxicity data, toxic potency, and considerations related to what technically may be achieved. The report also acknowledges the potential problem of unintended changes in non-regulated substances.

An issue specific to ST products is the extraction rate of different constituents. In contrast to food stuffs ST is not ingested, only a proportion of the content of potential toxicants is extracted from the product, which needs to be taken into account in a risk management analysis.

An important aspect when applying regulatory science to tobacco products is the availability of validated analytical techniques. In support of work to further develop standards for smokeless tobacco, proficiency testing of selected analytes was done at several laboratories in Europe coordinated by a working group under the auspices of the ESTOC Scientific Advisory Committee. This work is now continued and expanded by a newly formed CORESTA Subcommittee for ST products.

## Competing interests

All authors are or have been employees of Swedish Match AB.

## Authors' contributions

LER initiated and conceptualized the review, and wrote the paper. MC, TH, TR, and IW made substantial contributions to the acquisition of the presented data, provided factual information and background to the developments described, and were actively involved in drafting and revising the manuscript. MC also provided the internal documentation presented in the Table and in Figures 2 &3. All authors have read and given final approval to the submitted paper.
